# Corticospinal Tract Sparing in Cervical Spinal Cord Injury

**DOI:** 10.3390/jcm13216489

**Published:** 2024-10-29

**Authors:** Clarissa Pedrini Schuch, Lazar I. Jovanovic, Gustavo Balbinot

**Affiliations:** 1 MyantX Corp, Mississauga, ON L5K 2L1, Canada; clarissa.pedrini.schuch@myant.ca; 2 WearTech Labs, Simon Fraser University, Surrey, BC V3T 0M1, Canada; 3 Department of Biomedical Physiology and Kinesiology, Simon Fraser University, Burnaby, BC V5A 1S6, Canada; 4 Movement Neurorehabilitation and Neurorepair Laboratory, Simon Fraser University, Burnaby, BC V5A 1S6, Canada; 5 Institute for Neuroscience and Neurotechnology, Simon Fraser University, Burnaby, V5A 1S6 BC, Canada

**Keywords:** corticospinal tract integrity, sacral sparing, cervical spinal cord injuries, diagnostics, prognostics

## Abstract

Disruptions in the brain’s connections to the hands resulting from a cervical spinal cord injury (cSCI) can lead to severe and persistent functional impairments. The integrity of these connections is an important predictor of upper extremity recovery in stroke and may similarly act as a biomarker in cSCI. In this perspective article, we review recent findings from a large cohort of individuals with cSCI, demonstrating the predictive value of corticospinal tract (CST) integrity in cSCI—CST sparing. This research underscores that, akin to stroke, the integrity of brain-to-hand connections is crucial for predicting upper extremity recovery following cSCI. We address the limitations of commonly used metrics, such as sacral sparing and the concept of central cord syndrome. Furthermore, we offer insights on emerging metrics, such as tissue bridges, emphasizing their potential in assessing the integrity of brain connections to the spinal cord.

## 1. Introduction

Cervical spinal cord injuries (cSCI) have profound and debilitating effects on individuals, primarily due to the loss of sensorimotor function in muscles below the level of injury [1,2]. The extent of impairment varies with the severity of the lesion, with severe injuries potentially resulting in a complete loss of movement below the affected area. While neurorecovery typically occurs at or near the injury site, in some cases, recovery can extend to muscles distant from the lesion [3].

Muscles within the zone of partial preservation, which retain some degree of sensorimotor function despite being weakened, are generally more likely to regain function over time [4]. However, predicting which muscles will recover remains challenging with the current clinical assessments recommended by the International Standards for Neurological Classification of Spinal Cord Injury (ISNCSCI) [5]. This highlights the need for supplementary evaluations to better understand and predict the recovery potential of muscles impacted by cSCI.

A solution proposed through the European Multicenter Study about Spinal Cord Injury (EMSCI) [6] is the integration of electrophysiological multimodal assessments with traditional ISNCSCI evaluations. An international team recently demonstrated that these assessments provide a valuable complement to standard clinical evaluations by assessing the integrity of brain-to-hand connections. This pathway is crucial for transmitting sensorimotor information across anatomical spinal cord regions that are essential for the overall functionality of the sensorimotor system.

In this perspective article, we explore spinal cord anatomical regions—such as the integrity of lateral tract—through the concept of the eloquent spinal cord, which refers to areas crucial for maintaining normal sensorimotor function [7]. Regions with high functional eloquence are those where injury leads to significant deficits in function. Here, we advocate for testing these eloquent regions using electrophysiology to improve diagnostics and prognostics following cSCI. We discuss the application of motor evoked potentials (MEPs) to evaluate the integrity of the critical pathway to spinal cord regions under the umbrella of upper extremity neurorecovery—corticospinal tract (CST) sparing. We discuss how data obtained from this assessment can serve as an innovative biomarker, providing insights into the recovery potential of upper extremity muscles affected by cSCI beyond current metrics—sacral sparing [5]—and newly proposed metrics—midsagittal tissue bridges [8].

## 2. Segmental Motor Recovery After cSCI Relates to Density and Integrity of CST Projections

A recent study sheds light on the nuances of motor recovery following cSCI, revealing a proximal-to-distal gradient in motor recovery. Specifically, proximal muscles, such as those in the upper arm, demonstrated greater recovery potential compared to distal muscles in the hand. This pattern persists even when controlling for the distance from the injury site, suggesting an intrinsic difference in recovery capacity among muscle groups.

**Figure 1:**
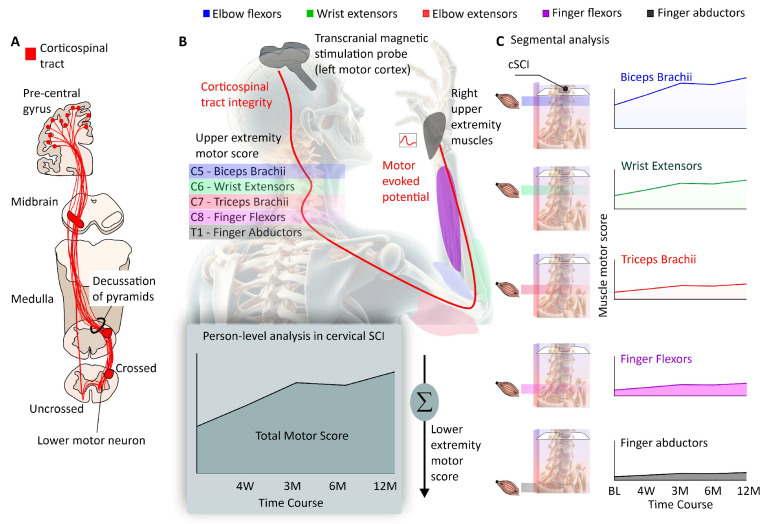
Corticospinal tract (CST) sparing and segmental analysis in cervical spinal cord injury. (**A**) The CST originates from the pre-central gyrus and extends to several spinal cord segments. (**B**) The integrity of the CST can be assessed by the motor evoked potential (MEP) using transcranial magnetic stimulation. Nonetheless, the ISNCSCI classification considers the summed residual strength of the upper and lower limbs (UEMS and LEMS) and sacral sparing—disregarding CST sparing. Segmental analysis refers to the study of the impairment and recovery of specific muscles affected by the cSCI and the quantification of CST sparing. This concept provides a nuanced understanding of muscle impairment and recovery potential. From a segmental analysis perspective, each muscle affected by cSCI is assessed individually, incorporating evaluations of CST sparing. (**C**) This analysis also includes the control of the distance from the specific myotome innervating the muscle and the motor level of the cSCI. It is crucial to assess muscle-specific characteristics in the spinal cord, such as reliance and degree of CST sparing and the number of lower motor neurons.

Observed differential recovery is hypothesized to be linked to the varying density of CST projections to different muscle groups. The CST, a critical pathway for voluntary motor control, has more extensive projections to hand muscles compared to proximal arm muscles. Consequently, when the CST is damaged in cSCI, hand muscles are disproportionately affected, leading to more severe impairment and limited recovery (Figure 1).

The results are based on a comprehensive analysis of data from 748 individuals living with cSCI collected over the course of EMSCI [6], which included ISNCSCI to assess muscle strength as well as electrophysiological measures, including MEPs, somatosensory evoked potentials, and nerve conduction studies.

**Figure 2:**
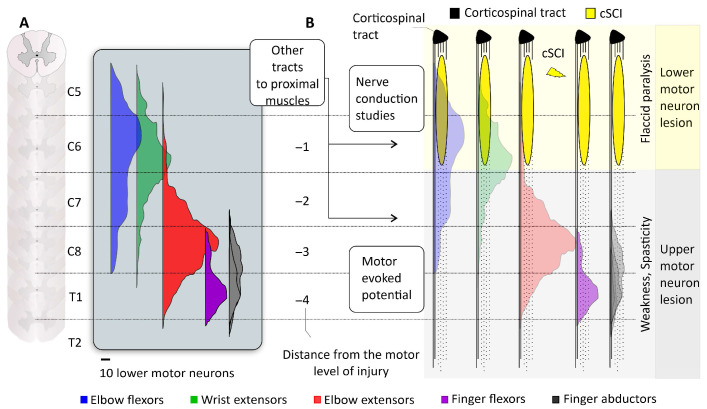
The anatomy and physiology supporting the segmental analysis in cervical spinal cord injury. (**A**) The recovery of muscle function depends on the sparing of the lower motor neurons innervating the muscle. The amount and distribution of lower motor neuron pools innervating different upper extremity muscles is represented in different colors (data extracted from non-human primates). The integrity of these pools can be evaluated through nerve conduction studies. (**B**) The segmental analysis takes into consideration anatomical and physiological factors such as the lesion size and location, the number of motor units innervating different upper extremity muscles, the reliance on corticospinal tract (CST) projections (or the possibility of compensation by other auxiliary tracts). Depending on the location of the lesion, different upper extremity muscles may present with flaccid paralysis (lower motor neuron syndrome) and/or spasticity, upper motor neuron syndrome. Electrophysiological assessments afford the diagnostics of these different syndromes and open opportunities for precision rehabilitation.

A key finding of the study is the predictive value of baseline muscle strength and MEPs in forecasting motor recovery, particularly for hand muscles. In individuals with more severe injuries (AIS A/B/C), the presence and amplitude of MEPs at baseline were significant indicators of potential recovery, aligning with the concept of CST integrity being crucial for motor recovery. Interestingly, individuals with positive MEPs at baseline recovered approximately 45% of lost upper extremity strength, a proportion lower than that observed in stroke recovery (about 70%). This difference underscores the unique challenges of cSCI recovery and the direct impact of CST damage. The study also explored the concept of proportional recovery, a framework widely used in stroke research. While some similarities were observed, particularly in less severe injuries (AIS D), the recovery patterns in cSCI showed greater variability and complexity. This variability highlights the complex nature of cSCI and suggests that factors beyond CST integrity, such as the segmental nature of spinal cord organization, may influence the recovery process (Figure 2). These findings not only emphasize the importance of preserved CST connections in cSCI recovery but also reveal the need for cSCI-specific models of recovery that account for the unique characteristics of spinal cord injuries.

From a clinical perspective, these findings have significant implications for prognosis, rehabilitation strategies, and clinical trial design in cSCI research. The ability to predict recovery potential based on early clinical and electrophysiological assessments, particularly MEPs, could enable more personalized and targeted rehabilitation approaches. By identifying muscles with greater recovery potential, clinicians could focus rehabilitation efforts more effectively, potentially optimizing outcomes. This aligns with the broader concept of the eloquent spinal cord, where certain regions are crucial for specific functions. The study’s demonstration of the prognostic value of MEPs, especially for hand muscle recovery, supports the use of electrophysiology in assessing these eloquent regions. Furthermore, the observed variability in recovery patterns and the importance of baseline CST integrity suggest that stratification based on early electrophysiological assessments could enhance the sensitivity of clinical trials and aid in patient selection. This approach could lead to more nuanced study designs, potentially improving the evaluation of new interventions and therapies for cSCI.

### Clinical Applications in the Field of Neuromodulation

Recently, it has been suggested that to fully grasp the therapeutic benefits of spinal cord stimulation, it is essential to consider the role of intrinsic corticospinal inputs. Research utilizing advanced biophysical models of motoneuron membrane behavior showed that spinal cord stimulation can deliver sensory inputs below the threshold of activation, which are then converted into supra-threshold responses when combined with voluntary drive [9]. These model predictions have been confirmed through electrophysiological studies conducted on monkeys, stroke patients, and individuals with spinal cord injuries. Similarly, a groundbreaking brain–spine interface utilized electrocorticographic techniques to measure activity from the sensorimotor cortex. It enabled the transmission of intended movement to the spinal cord—allowing an individual with chronic tetraplegia to stand and walk naturally in community settings [10,11]. This advancement represents a significant leap forward in rehabilitation technology, enhancing mobility and quality of life for those affected. This underscores the critical importance of the information transmitted through the CST.

Building on these insights, brain–computer interface-controlled functional electrical stimulation therapy emerges as a promising rehabilitation strategy for targeted and segmental motor recovery after cSCI. Since the Pfurtscheller and colleagues [12] demonstration of the feasibility of brain–computer interface-controlled functional electrical stimulation therapy in restoring upper limb motor function, the approach has evolved from an assistive device to a therapeutic tool [13,14]. Due to the modular nature of functional electrical stimulation devices [15], brain–computer interface-controlled functional electrical stimulation therapy systems can now be tailored to facilitate recovery of both proximal and distal muscles, depending on individual patient needs and therapist assessments [16]. This flexibility aligns well with the observed proximal-to-distal gradient in motor recovery and the varying CST projection densities to different muscle groups. Moreover, by incorporating MEP response data from individual muscles, functional electrical stimulation can be further refined to target muscles with the highest recovery potential, as indicated by the study’s findings on the predictive value of baseline MEPs. This personalized approach to rehabilitation resonates with the concept of the eloquent spinal cord and the need for cSCI-specific recovery models, potentially offering a more effective strategy for addressing the complex and variable nature of motor recovery in cSCI.

## 3. The Eloquent Spinal Cord: Lesion-Affected and Recovery-Related Networks

**Figure 3:**
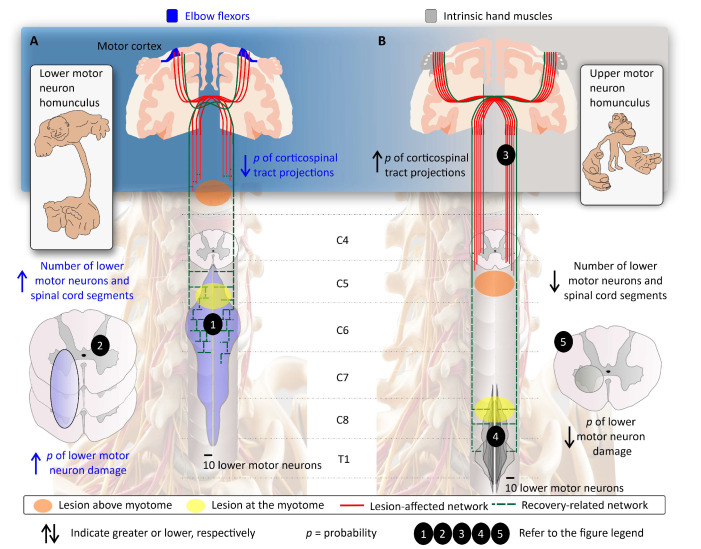
Lesion-affected and recovery-related networks and segmental recovery. Lesion-affected (red lines) and recovery-related (green lines) networks are represented in a cervical lesion where the muscle-specific myotome is 3 spinal cord segments below the cSCI (orange lesion) and at the myotome (yellow lesion). (**A**, **upper panels**) The lower motor neuron homunculus highlights the greater proportion of lower motor neurons dedicated to proximal upper extremity muscles compared to distal hand muscles [19]. (**B**, **upper panels**) The upper motor neuron homunculus depicts the greater proportion of upper motor neurons dedicated to distal hand muscles compared to proximal upper extremity muscles [20,21]. (**A**, **bottom panels**) In cervical spinal cord injury (cSCI), a severe incomplete lesion at the C3 level (orange) will lead to loss of corticospinal tract (CST) integrity (lesion-affected network; red lines). Proximal upper limb muscles have a higher concentration of lower motor neurons [22] but fewer direct CST projections [23,24] compared to distal hand muscles. The recovery-related network for elbow flexors will include the residual CST sparing (indicated by green hashed lines) (**1**), which undergoes plasticity to reinnervate remaining—and abundant—lower motor neuron pools below the injury level. A similar lesion at the C5/C6 level will result in additional lower motor neuron loss. However, because these motor neurons span several spinal cord levels and are numerous (as seen with elbow flexors in this example) (**2**), it will generate a favorable recovery prognosis, akin to what is observed clinically. (**B**, **bottom panels**) In a similar lesion, the segmental recovery of distal hand muscles may vary significantly. The proximity of a muscle’s myotome to the motor level of the spinal cord injury plays a significant role in recovery outcomes. Recovery is typically more pronounced in muscles located at or near the injury level. The lesion (indicated in orange) is much farther from the lower motor neurons, which challenges the limited neuroplasticity of CST axons [25,26]. Secondly, the great reliance of hand muscles on CST projections (**3**) and the limited number of lower motor neurons per muscle (**4**) makes the distal hand muscles very susceptible to the lesion. A less common type of lesion affecting the C8 spinal segment (indicated in yellow) may also cause lower motor neuron damage. The segmental distribution in the spinal cord differs: proximal muscles are associated with a broader segmental range, while distal muscles are concentrated in specific segments (C8-T1) [27]. Thereby, lower motor neuron lesions are less likely for hand muscles given the limited number of spinal segments and lower motor neurons spanning the cross-sectional area of each segment that innervate the intrinsic hand muscles (**5**).

The lesion-affected network often encompasses eloquent spinal cord regions—areas crucial for functional recovery [7]. Research, including our own, highlights the significance of preserving lateral tracts for recovery prognosis after spinal cord injury [6,17,18]—the recovery-related networks (Figure 3). For instance, our study demonstrated that CST integrity early after cSCI is a key predictor for whether hand muscles will regain strength one year after injury [6], identifying the lateral tract as an eloquent region for hand function recovery [7].

Similarly, for lower-limb muscles, motor synergy encoding neurons are critical for locomotion and are located in the intermediate and ventral layers of the spinal cord [28]. The activation of these synergy encoding neurons can be measured using electrophysiology, showing patterned activation during muscle spasms or with spinal cord stimulation [29]. These neurons play an essential role in gait function after spinal cord injury and are the building blocks of what we know as central pattern generators. Little is known about these generators in the cervical cord but flexor and extensor patterns are evident in upper extremity muscles, which are affected by cSCI and stroke [30,31,32]. We propose that transcutaneous cervical spinal cord stimulation will pave the way in the coming years for a deeper understanding of pattern generators—motor synergy encoding neurons integrity—in the cervical cord [33,34]. Recruitment curves obtained from cervical spinal cord stimulation might uncover residual synergistic activity in upper limb muscles, suggesting the presence of preserved motor synergy encoding neurons. This is similar to the induction of rhythmic locomotor activity with spinal cord stimulation of the lumbar region [29].

The integrity of the Rexed lamina IX, which contains lower motor neurons that innervate muscle fibers (i.e., motor units), can be assessed by electromyography [35] or nerve conduction studies [36]. Diagnosing a lesion in the lower motor neuron is crucial, as restoring or regenerating the long connection from the neuron body to the muscle is unlikely. This diagnosis may indicate the need for nerve transfer surgery [37]. For example, in cases of a C5 spinal cord lesion, assessing lower motor neuron integrity through nerve conduction studies of the musculocutaneous nerve is crucial. This evaluation helps determine the impairment and recovery potential of proximal muscles such as the *deltoid* and *biceps brachii*. Examining CST sparing through MEPs in hand muscles is beneficial, given the potential impact of a C5 lesion on descending CST pathways to hand muscles. To test the integrity of connections within spinal cord segments, transcutaneous or epidural spinal cord stimulation can indicate the degree of preservation in intermediate spinal cord laminae containing motor synergy encoding neurons [29,33,34]. Accurate synergistic action of upper extremity muscles is essential for proper hand and arm function.

**Figure 4:**
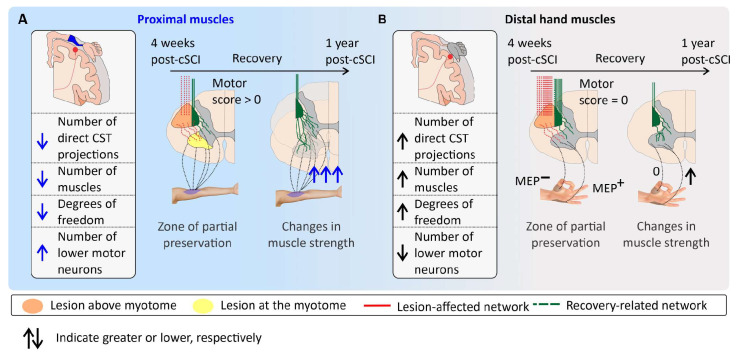
Mechanisms of segmental recovery in cervical spinal cord injury (cSCI): insights from residual strength and corticospinal tract (CST) sparing. (**A**) Segmental recovery in the scenarios described in Figure 3A: residual strength is detected in the ISNCSCI given the large amount of lower motor neurons innervating proximal muscles, which even with limited CST sparing can generate tetanus (Motor score > 0). Recovery will activate recovery-related networks driven by residual CST sparing, leading to the reinnervation of numerous lower motor neurons below the injury level—potentially by engaging relay neurons in the intermediate Rexed laminae. Changes in muscle strength 1-year after cSCI are significant. (**B**) Segmental recovery in the scenarios described in Figure 3B: the absence of residual strength in the ISNCSCI is due to the heavy reliance of intrinsic hand muscles on CST sparing and the limited number of lower motor neurons per muscle. However, electrophysiological assessments can reveal motor evoked potentials (MEPs) in some muscles, indicating CST sparing. With intensive neurorehabilitation, recovery of these MEP^+^ muscles can be observed one year after cSCI, reflecting targeted plasticity of CST projections. Muscles without early CST sparing (MEP^−^) show no recovery.

Electrophysiological multimodal assessments, thereby, are ideally suited to evaluate the sparing of eloquent spinal cord regions, offering valuable diagnostic and prognostic insights after cSCI [38]. Electrophysiological multimodal assessments should be tailored to each individual’s injury level to accurately address the affected and recovery-related networks (Figure 4). This approach marks a significant advancement in cSCI research, addressing the neuroanatomical–functional paradox [7] and segmental recovery [6,39] by providing a more nuanced perspective on the lesion-affected and recovery-related networks.

## 4. Corticospinal Tract (CST) Sparing: Optimizing Diagnostics and Prognostics in Cervical Spinal Cord Injuries (cSCI)

### 4.1. Central Cord Syndrome

In the spinal cord, the anatomical location of CST projections to specific muscles—legs lateral, trunk intermedial, arm medial—has long been a fundamental concept within the scientific community and is a key component of many textbooks [40]. Nevertheless, it was not until recently that this theory was challenged by well-designed experiments conducted by the teams of Roger N. Lemon and Robert J. M. Morecraft [41]. Using the non-human primate model, the authors demonstrated that the previously assumed somatotopic organization of descending CST projections is not accurate.

This study conclusively refutes the notion of somatotopy among CST fibers traversing the lateral CST, in contrast to the well-established somatotopy found in the cortex, corona radiata, and internal capsule. The findings indicate that CST fibers in the eloquent spinal cord are uniformly susceptible to both focal and diffuse injuries, regardless of their cortical origin. As a result, the marked impairment of arm and hand movements following cSCI, usually termed as a central cord syndrome lesion, is likely attributed to factors beyond somatotopy, such as the heightened dependence of hand and arm functions on the CST compared to the lower limbs [42,43]. This complicates the use of imaging techniques to distinguish projections to hand muscles from other CST projections.

### 4.2. Midsaggittal Tissue Bridges

A promising direction in this field is the exploration of tissue bridges and their role in quantifying CST integrity. In a recent study using midsagittal tissue bridges, diffusion tensor imaging metrics were discussed and recognized for their potential benefits. However, limitations were noted, including challenges at the lesion site due to metal-induced artifacts and limited resolution [8]. The authors argue that further technical advancements are required to improve its efficacy and that future research should focus on evaluating parasagittal slices to gain a clearer understanding of the extent and location of preserved tissue. The parasagittal tissue bridge is more likely to contain reliable information about lateral tract integrity but follow up studies are necessary to determine its utility.

Applying this approach to evaluate parasagittal tissue bridges in cSCI could offer new insights into the relationship between CST integrity and recovery potential. This perspective aligns with findings from many studies in the stroke field that use magnetic resonance imaging to analyze stroke lesion overlap and quantify CST integrity—demonstrating its effectiveness in predicting functional outcomes [44,45,46,47,48,49,50].

The concept of tissue bridges is appealing in terms of quantifying CST integrity. As previously mentioned, similar approaches have been extensively researched in stroke studies. For instance, fractional anisotropy measurements, obtained through diffusion tensor imaging techniques, have demonstrated a correlation between CST integrity and long-term hand function prognosis [51]. It is also possible to use magnetic resonance imaging to measure CST-weighted lesion load without the use of diffusion tensor imaging techniques. In stroke, findings suggest that diffusion tensor imaging methods are not needed to gain a precise predictor of upper limb motor outcomes [52]. Lesion-load metrics can be calculated from the lesion volume in the T2-image overlaid on the CST map from the standard template [53]. Additionally, higher resolution in magnetic resonance imaging can be achieved through longer acquisition times and the use of advanced magnetic resonance imaging technology. In the stroke field, several metrics have been explored to assess lesion overlap with the CST, with the most effective ones being those that measure the degree of overlap. Metrics such as maximum overlap and percent subsections have proven to be superior to others in evaluating this relationship [53]. Therefore, accurately quantifying lesion extent and overlaying it with spinal cord templates represents a promising direction for advancing research on tissue bridges in cSCI.

The current limitations in using tissue bridges for cSCI, combined with the understanding that the CST lacks clear somatotopy, suggest that electrophysiological measures of CST integrity may currently be more reliable indicators of CST integrity.

### 4.3. Corticospinal Tract (CST) Sparing: Motor Evoked Potential (MEP)

Recent advancements in understanding cSCI and stroke rehabilitation have highlighted several key concepts in evaluating and predicting recovery. Segmental prediction articles have explored how various levels of spinal cord injury affect functional outcomes [6,39], but the concept of CST sparing remains central to these discussions. The lack of clear somatotopic organization within the CST complicates the use of anatomical landmarks for predicting recovery—using, for example, tissue bridges obtained from magnetic resonance imaging [8]. In this section, we will explore electrophysiological methods for quantifying CST sparing, emphasizing its critical role as a key predictor of upper extremity recovery [6]. This concept has gained prominence due to its alignment with research findings in both stroke and cSCI studies [6,45,49,54].

CST sparing has been extensively evaluated using electrophysiological techniques, such as MEPs. In stroke research, the relationship between metrics obtained from magnetic resonance imaging, specifically using diffusion tensor imaging techniques (like fractional anisotropy) and electrophysiology have been examined within the framework of CST sparing and the proportional recovery rule [54,55,56]. Diffusion-weighted magnetic resonance imaging allows for the evaluation of disruptions in the microstructural organization of white matter, using measures such as fractional anisotropy and axial diffusivity [57]. Given that these metrics are complicated in cSCI due to the aforementioned metal-induced artifacts [8], an alternative solution is the use of electrophysiological biomarkers, such as motor evoked responses obtained through transcranial magnetic stimulation.

Given the extensive brain region involved in encoding and generating dexterous hand movements [20,21], the established methodologies [58], and the absence of artifacts from metal implants used in decompression surgery, this method appears ideal for assessing CST sparing. By focusing on the source of efferent signals from the brain to the hands, this approach also addresses the challenge of the lack of somatotopy in CST projections to upper extremity muscles in the spinal cord [41,42].

We have recently explored the predictive value of CST sparing for recovery prognostics after cSCI using machine learning techniques [6]. The MEP early after the injury (within the first 4 weeks after the injury) was a strong predictor of upper extremity recovery 1-year after cSCI. From this perspective, and given the challenges in establishing ideal magnetic resonance imaging methods for quantifying CST sparing [8], it is reasonable to consider MEPs as the primary method [6].

Understanding the recovery prognosis of different upper extremity muscles early after a cSCI is crucial, as it allows for the targeted and intensive rehabilitation of muscle groups with an anticipated positive recovery outlook. This can be achieved through mass practice during activity-based therapy [59] and combined with innovative and promising treatments to enhance neurorecovery, such as anti-NOGO antibody therapy [60,61], stem cell therapy [62], spinal cord stimulation [63], and functional electrical stimulation therapy—with or without brain–computer interface control [64,65]. Similarly to stroke, accurate prediction of muscle recovery potential enables the timely application of these novel therapies during the optimal neuroplasticity window following neurological damage, thereby maximizing their therapeutic effects [66].

**Figure 5:**
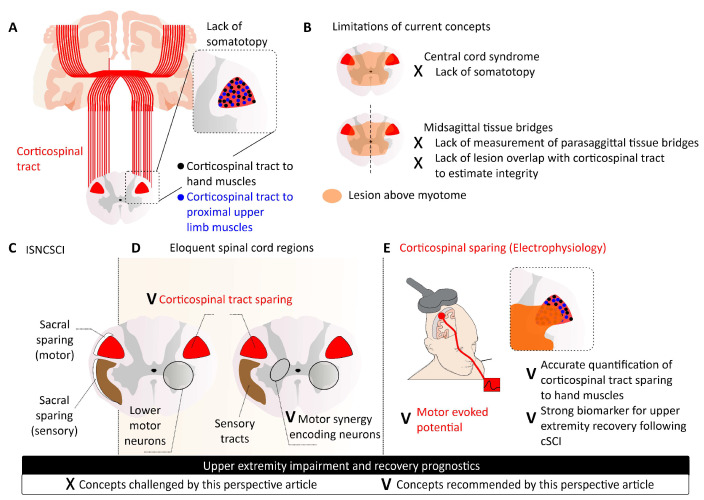
Corticospinal tract (CST) sparing and eloquent spinal cord regions. (**A**) Absence of clear somatotopy in CST projections [41]. (**B**) Limitations of the concept of central cord syndrome [43] and sagittal tissue bridges [8], emphasizing the need for diffusion tensor imaging or lesion overlap analysis. (**C**) Traditional concepts of sacral sparing do not account for the variability observed in upper extremity recovery after spinal cord injury [6]. (**D**) Electrophysiological testing of eloquent spinal cord regions, including motor evoked potentials (MEPs) for CST sparing, somatosensory evoked potentials for sensory tract sparing, and assessments of lower motor neurons (nerve conduction studies) and motor synergy encoding neurons (cervical spinal cord stimulation) for grey matter sparing, to enhance diagnostic and prognostic evaluations following cervical spinal cord injury (cSCI). (**E**) CST sparing and key spinal cord regions are critical for diagnosis and prognosis after cSCI. While CST projections lack somatotopy and sagittal tissue bridges have limitations, electrophysiological testing—such as MEPs for CST sparing—can provide valuable insights.

For example, lateral tract sparing has been implicated in in spasticity and recovery after SCI [17,18] and the residual upper neuron commands deemed necessary for the effectiveness of cervical spinal cord stimulation for upper extremity neurorecovery [9]. By integrating the concept of CST sparing in cSCI, we can better understand and predict recovery trajectories for individuals, moving beyond traditional metrics to more accurate assessments of neuroplasticity and functional recovery (Figure 5).

### 4.4. Limitations: Proposed and Current Techniques and Assessments

The ISNCSCI offers practical advantages over the proposed techniques. It is time-efficient, well established, and validated. In contrast, the proposed techniques require time-consuming acquisition protocols and extensive data analysis and processing. For example, electrophysiological assessments, such as the MEP, require the precise placement of electrodes on muscles and stimulation using magnetic coils positioned over specific locations on the precentral gyrus. Similarly, magnetic resonance imaging can involve extended acquisition times and expensive equipment, and it is often complicated by artifacts from metal components used to stabilize the spine during decompression surgery. Technological advancements are expected to enable broader use of these techniques in the future, contributing to improved diagnostics and prognostics in cSCI.

## 5. Conclusions and Future Directions

We conclude this perspective with several key takeaways. The concepts of eloquent spinal cord regions and lesion-affected and recovery-related networks must be embraced. We suggest that employing electrophysiological multimodal assessments is essential in this context, with significant implications for both diagnosis and prognosis following cSCI [7]. Segmental analysis will link specific spinal cord circuitry to muscle function, allowing for a more precise evaluation of each muscle and associated function [39]. The MEP has the selectivity needed to probe the integrity of the critical spinal cord regions for upper extremity recovery—brain-to-hand connections, i.e., CST sparing [6]. Innovative methods for assessing tract and circuitry sparing after cSCI are needed, such as the previously mentioned use of spinal cord stimulation to evaluate the integrity of motor synergy encoding neurons. Discovering new ways to explore recovery-related networks, combined with detailed segmental analysis, will propel advancements in the field over the coming decades.

## Abbreviations

cSCI = cervical spinal cord injury; ISNCSCI = international standards for neurological classification of spinal cord injury; CST = corticospinal tract; AIS = ASIA impairment scale; MEP = motor evoked potential; C = cervical; T = thoracic; BL = baseline; W = week; M = month; UEMS = upper extremity motor score; LEMS = lower extremity motor score.
